# Allergic reactions to propofol in adult patients with egg or soybean allergy: a retrospective cohort study from a large database of a single institute

**DOI:** 10.1186/s40981-022-00591-8

**Published:** 2023-01-09

**Authors:** Masaki Iwakiri, Reo Inoue, Kanji Uchida

**Affiliations:** Department of Anesthesiology and Pain Relief Center, The University of Tokyo Hospital, 7-3-1, Hongo, Bunkyo-ku, Tokyo, 113-8655 Japan

**Keywords:** Propofol, Egg allergy, Soybean allergy, Drug allergy

## Abstract

**Background:**

In recent years, many reports have indicated that propofol is safe to administer to patients with egg/soybean allergy in Western countries. Egg allergy is more frequent in Asia, but there are limited reports regarding allergic reactions to propofol use among adults. This study aimed to determine whether propofol causes allergic reactions in patients with egg/soybean allergy.

**Methods:**

Adult patients who underwent surgery involving anesthesiologists from 2018 to 2021 were included. In all patients, we reviewed food allergy information in their electronic medical record and extracted anesthetics. Patients with egg/soybean allergy were subdivided into two groups on the basis of intraoperative use of propofol. We evaluated each group for allergic reactions within 24 h after the induction of anesthesia. The primary outcome was a relative risk of allergic reactions after propofol use for patients with egg/soybean allergy.

**Results:**

In total, 22,111 patients with 28,710 anesthesia records were identified. Among patients with egg/soybean allergy, 173 (0.8%) patients and 237 (0.8%) anesthesia records were included in the study. Among the records of egg-/soybean-allergic patients, 151 were administered propofol, and 86 were not. The relative risk of allergic reactions after propofol use for patients with egg/soybean allergy was 1.14 (95% confidence interval, 0.10–12.4; *p* = 0.74).

**Conclusion:**

The use of propofol in patients with egg/soybean allergy does not significantly increase the relative risk of allergic reactions. Therefore, anesthesiologists can appropriately determine the indication for propofol, even in patients with egg/soybean allergy.

**Trial registration:**

UMIN-CTN, UMIN000049321 registered 26 October 2022 — retrospectively registered, https://center6.umin.ac.jp/cgi-open-bin/ctr/ctr_view.cgi?recptno=R000056167

**Supplementary Information:**

The online version contains supplementary material available at 10.1186/s40981-022-00591-8.

## Background

An allergic reaction to anesthetics, including anaphylaxis, is a serious perioperative complication. To avoid an allergic reaction, anesthesiologists refer to the patient’s allergic history when selecting the anesthetic agent [1].

Propofol is a common anesthetic in Japan. The incidence of anaphylaxis due to propofol is 1 in 60,000 cases, and 1.2% of instances of perioperative anaphylaxis are caused by propofol in France [2]. Propofol induces anaphylaxis less frequently than muscle relaxants, sugammadex, and antibiotics [3]. There is some debate regarding the safety of administering propofol to patients with a food allergy to egg/soybean because it contains egg yolk lecithin and soybean oil [4]. In addition, there have been several reports of propofol causing anaphylaxis in patients with egg/soybean allergy [5–7]. However, Murphy et al. showed that propofol is safe for most egg-allergic children without a history of anaphylaxis to eggs [8]. The drug guideline published by the Japanese Society of Anesthesiologists mentions positive and negative opinions regarding the relationship between propofol and egg/soybean allergy [9].

The prevalence of food allergies is influenced by geographical locations and feeding patterns [10]. The prevalence of egg allergy ranged from 3 to 4% in a Chinese food challenge-proven study [11] and 3.2 to 5.3% in a Japanese caregiver-reported study [12]. These rates are higher than the Western challenged-proven prevalence of egg allergy ranging from 1 to 1.6% [11]. However, to date, no large-scale study has investigated the use of propofol in egg-/soybean-allergic patients and the frequency of allergic reactions in Japan. Therefore, this study aimed to determine whether propofol causes allergic reactions in Japanese patients with egg/soybean allergy.

## Methods

This retrospective cohort study was approved by the University of Tokyo Clinical Research Review Board (approval number: 2203-(7)). The study was carried out after an opt-out period announced on the institute’s website to allow patients to refuse participation. The need to obtain written informed consent from each patient was waived.

Using anesthesia records, we identified adult patients (≥ 20 years of age) who underwent surgery involving anesthesiologists from 1 January 2018 to 31 December 2021. Electroconvulsive therapy was excluded from this study because the protocol is fixed, and propofol is never used for this therapy at our institution. We checked the electronic medical records of all patients for egg/soybean allergy. Electronic medical records include the history of food service during hospitalization; therefore, food allergies are always described. The data of age, sex, method of anesthesia, and the American Society of Anesthesiologists physical status classification (ASA-PS) were extracted from the anesthesia records.

Patients with egg/soybean allergy were subdivided into two groups on the basis of the intraoperative use of propofol. We evaluated each group for allergic reactions within 24 h after the induction of anesthesia. Any possibility of an allergic reaction was considered if one of the following criteria was met: a written comment regarding a specific allergic sign (e.g., skin rash), the mention of a suspicion of an allergic reaction on their anesthesia records or postoperative notes, or the administration of antihistamines, corticosteroids, or epinephrine during surgery or the postoperative period. Pretreatment with antihistamines or corticosteroids was also reviewed because these drugs might mask a minor allergic reaction.

The primary outcome was a relative risk of allergic reactions after propofol use compared with other anesthetics among patients with egg/soybean allergy.

To calculate the sample size, we estimated that the incidence rate of allergic reactions in patients without intraoperative propofol use was 0.1% in accordance with a previous report [1]. Assuming that the use of propofol is associated with a high risk of allergic reactions in patients with egg/soybean allergy, the incidence of allergic reactions caused by propofol in these patients was estimated to be 10%. The significance level alpha was 5%, and the power was 80%. In this setting, the sample size was 80 cases in each group.

Continuous variables are shown as means and standard deviations, and categorical variables are shown as frequencies and proportions. The chi-square test, Fisher’s exact test, and *t*-test were used for comparison between groups, and *p* < 0.05 was considered significant. In patients with egg/soybean allergy, relative risks of allergic reactions were calculated. The sample size was calculated using EZR 1.55 (Saitama Medical Center, Jichi Medical University, Saitama, Japan) [13], which is a graphical user interface for R (the R Foundation for Statistical Computing, Vienna, Austria). Other analyses were conducted using JMP Pro 16.1.0 (Cary, NC, USA).

## Results

**Fig. 1:**
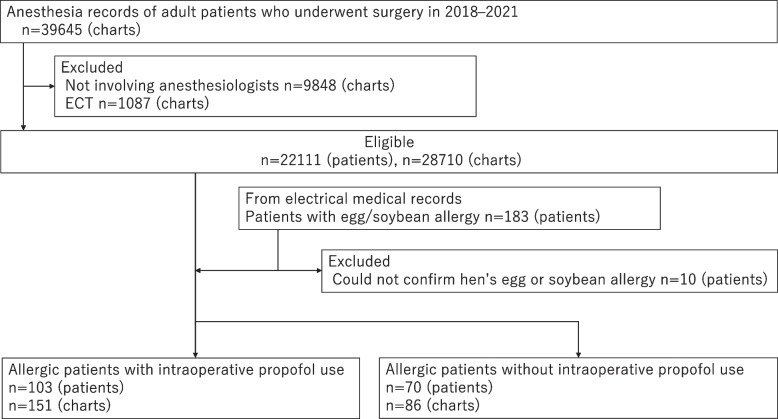
Flowchart of the study. ECT, electroconvulsive therapy

In total, 22,111 patients and 28,710 anesthesia records were identified. In patients with egg/soybean allergy, 173 (0.8%) patients and 237 (0.8%) anesthesia records were analyzed (Fig. 1).

Details of the preoperative and intraoperative anti-allergic medications are shown in Table 1.

**Table 1:** Preoperative and intraoperative anti-allergic medications

	With intraoperative propofol use (*n* = 151)	Without intraoperative propofol use (*n* = 86)	*p*
Age	47.0 (1.5)	50.0 (2.0)	0.24
Male	49 (32.5%)	20 (23.3%)	0.14
Allergy			
Egg	140 (92.7%)	65 (75.6%)	0.0003
Soybean	9 (1.3%)	19 (22.1%)	0.0005
Both	2 (1.3%)	2 (2.3%)	0.62
Corticosteroids	34 (22.5%)	20 (23.3%)	1
Preoperative	17 (11.3%)	13 (15.1%)	0.42
Intraoperative	19 (12.6%)	7 (8.1%)	0.39
Both	2 (1.3%)	0	0.54
Antihistamines	27 (17.9%)	23 (26.7%)	0.14
Preoperative	26 (17.2%)	17 (19.8%)	0.73
Intraoperative	1 (0.7%)	7 (8.1%)	0.0053
Both	0	1 (1.2%)	0.36
Epinephrine	2 (1.3%)	0	0.54
Preoperative	0	0	-
Intraoperative	2 (1.3%)	0	0.54

Continuous variables are described as mean (standard deviation)

The group of patients with propofol use contained more patients with egg allergy (*p* = 0.0003), and the other group of patients without propofol use contained more patients with soybean allergy (*p* = 0.0005). Corticosteroids, antihistamines, and epinephrine were administered during the operative period in 34 (22.5%), 27 (17.9%), and 2 (1.3%) patients with intraoperative propofol use and in 20 (23.3%), 23 (26.7%), and 0 patients without intraoperative propofol use, respectively. Intraoperative antihistamines were administered in significantly more patients without intraoperative propofol use than in those with intraoperative propofol use (*p* = 0.0053). The most common reason for preoperative steroid administration was to prevent an asthmatic attack. The most common reason for intraoperative steroid administration was to prevent postoperative nausea and vomiting. In this study, intraoperative steroids and antihistamines were not used to treat allergic reactions in any patient. Intraoperative epinephrine was administered as an inotrope in one patient and as a treatment for anaphylaxis in another patient.

Regarding allergic reactions, one case of skin rash and one case of anaphylaxis were observed in patients with intraoperative propofol use and one case of skin rash in those without intraoperative propofol use (2/151 vs. 1/86). The relative risk of allergic reactions after propofol use in patients with egg/soybean allergy was 1.14 (95% confidence interval, 0.10–12.4; *p* = 0.74).

One patient with anaphylaxis was observed in the intraoperative propofol use group. The patient was an 80-year-old male with a height of 160 cm and a weight of 54 kg. His ASA-PS was 2E. He received general anesthesia with propofol. During induction of anesthesia with propofol, problems did not occur for him. However, he developed severe hypotension and skin erythema intraoperatively. Immediately he was diagnosed and treated for anaphylactic shock with adrenaline. The surgery was completed, and he was followed up in the intensive care unit; no new allergic reactions occurred. Ketamine, concentrated red blood cell transfusion, and antibiotics (piperacillin/tazobactam) were suspected as causative factors for anaphylaxis, based on the timing of the anaphylaxis.

## Discussion

Using anesthesia records, we investigated whether propofol causes allergic reactions in patients with egg/soybean allergy. Propofol use was not significantly associated with a higher relative risk of allergic reactions in patients with egg/soybean allergy.

In this study, 0.8% of patients who underwent surgery involving anesthesiologists were allergic to egg/soybean (supplemental Table 1). In Japan, the prevalence of food allergy is reported to be 1–2% in all age groups. Approximately 35% of food allergies are reportedly caused by eggs and approximately 2% by soybeans, which is consistent with the prevalence found in this study [14].

Propofol contains soybean oil and egg yolk lecithin [4]. There is some controversy regarding the safety of administering propofol to patients with egg/soybean allergy. However, in recent years, many reports have indicated that propofol can be used safely in patients with food allergies [8, 15–17]. Egg yolk lecithin is not a major egg allergen, and allergic reactions to it are considered rare [18]. Regarding soybeans, propofol contains soybean oil, but soybean-derived proteins, which can cause allergies, have been removed. Therefore, there is no need to avoid propofol administration in patients with soybean allergy [15]. Indeed, our study supports the notion that, in Japanese clinical practice, propofol can be safely used in patients with egg/soybean allergy.

In our study, in patients who maintained anesthesia with inhaled anesthetics, thiopental was used as an induction agent more frequently than propofol in patients with egg/soybean allergy (supplemental Table 2). This finding suggested that some anesthesiologists tended not to use propofol in patients with egg/soybean allergy. However, the frequency of total intravenous anesthesia with propofol was not affected by the presence of a food allergy. This finding indicated that propofol was used in cases where the benefits of total intravenous anesthesia were obvious, such as in craniotomies [19], surgeries requiring motor-evoked potential monitoring [20], and in patients with a high risk for postoperative nausea and vomiting [21]. The factors used by anesthesiologists to decide whether to use propofol in patients with egg/soybean allergy are unclear. More cases need to be accumulated to determine these factors.

The frequency of allergy to propofol in patients with egg/soybean allergies in Japan is unclear. We assumed that more anesthesiologists would not administer propofol to patients with egg/soybean allergies if the frequency of allergic reactions to propofol was at least 10%. Therefore, the sample size in this study was calculated assuming a 10% frequency of allergic reactions to propofol in patients with a food allergy. We found that in the group of patients with egg/soybean allergy, the rate of allergic reactions to propofol was 1.32%, and the relative risk of allergic reactions after propofol use for patients with egg/soybean allergy was 1.14 (95% confidence interval, 0.10–12.4). On the basis of these results, we estimate that the actual frequency of allergic reactions is only a few percent when propofol is used in patients with food allergies. Clarification of the relative risk by accumulating and analyzing clinical use experiences is important in the future. The true relative risk would allow anesthesiologists to make better clinical decisions when selecting anesthetics for patients with egg/soybean allergies.

There are several limitations to this study. First, because this was a retrospective cohort study, there may have been unmeasured confounders or inherent biases. Severe asthma, atopic complications, or a history of anaphylaxis may have influenced the decision of anesthesiologists on whether to use propofol. Because of the nature of this study as a retrospective chart review analysis, investigation of the factors that individual anesthesiologists considered important in their choice of anesthetic agents was not possible. Second, this study is underpowered to determine the true relative risk of allergic reactions after propofol use compared with other anesthetics among patients with egg/soybean allergy. To examine whether propofol should be avoided clinically, the sample size is calculated assuming a 10% incidence of allergic reactions to propofol in patients with egg/soybean allergies. However, the observed incidence of allergy in the group of patients who used propofol was lower than estimated. Therefore, a large clinical study based on the incidence obtained in this study is needed to determine the true relative risk. Third, whether a patient has a food allergy is based solely on the patient’s declaration. Because antibody measurements and skin tests were not performed, there may have been cases in which food allergies had already resolved. However, by referring to self-reports and the food served during hospitalization, patients who reported having a food allergy were identified as those who routinely avoid particular food. Fourth, we did not consider the severity of egg/soybean allergy. Therefore, we cannot conclude whether the use of propofol for patients who previously experienced egg/soybean-induced anaphylaxis is safe. Fifth, minor allergic reactions that were not clinically problematic may have been overlooked. In this study, allergic reactions were identified by referring to the records of operating room nurses, ward nurses and surgeons, and those recorded by anesthesiologists. By referring to judgments made by more than one person, this should have prevented allergic reactions from being overlooked.

## Conclusion

This study shows that the use of propofol in patients with egg/soybean allergy does not significantly increase the relative risk of allergic reactions after propofol use in patients with egg/soybean allergy. Anesthesiologists can appropriately determine the indication for propofol, even in patients with egg/soybean allergy.

## Supplementary Information

**Additional file 1: Supplemental Table 1.** Demographics of patients with or without egg/soybean allergy. **Supplemental Table 2.** Demographics from anesthesia charts of patients with or without egg/soybean allergy.

## Data Availability

The datasets used and/or analyzed during the current study are available from the corresponding author on reasonable request.
