# Supplementary material for: Allergic reactions to propofol in adult patients with egg or soybean allergy: a retrospective cohort study from a large database of a single institute
Source: JA Clin Rep. 2023 Jan 9;9:1. doi: 10.1186/s40981-022-00591-8 (PMC9826766; doi:10.1186/s40981-022-00591-8)
Supplement: Supplementary file 1 — Additional file 1: Supplemental Table 1. Demographics of patients with or without egg/soybean allergy. Supplemental Table 2. Demographics from anesthesia charts of patients with or without egg/soybean allergy. [file 40981_2022_591_MOESM1_ESM.docx]

**Supplemental Data**

Supplemental table 1. Demographics of patients with or without egg/soybean allergy.

|  | With egg/soybean allergy  (n=173) | Without egg/soybean allergy  (n=21938) | p |
| --- | --- | --- | --- |
| Age (year) | 49.7 (1.34) | 57.8 (0.12) | <0.001 |
| Sex (male) | 47 (27.2%) | 10172 (46.4%) | <0.001 |
| Egg allergy^¶^ | 153 (88.4%) | 0 |  |
| Raw eggs | 81 (52.9%)* | 0 |  |
| Soybean allergy^¶^ | 23 (13.3%) | 0 |  |

*Percentage of patients with allergy to raw eggs among those with egg allergy.

^¶^Four (2.3%) patients with both egg and soybean allergies.

Supplemental table 2. Demographics from anesthesia charts of patients with or without egg/soybean allergy.

| With egg/soybean allergy  (n=237) | Without egg/soybean allergy  (n=28473) | p |  |
| --- | --- | --- | --- |
| ASA-PS |  |  | 0.87 |
| 1 & 1E | 71 (30.0%) | 8185 (28.8%) |  |
| 2 & 2E | 124 (52.3%) | 15901 (55.9%) |  |
| 3 & 3E | 37 (15.6%) | 3724 (13.1%) |  |
| 4 & 4E | 4 (1.7%) | 586 (2.1%) |  |
| 5 & 5E | 0 (0%) | 6 (0.021%) |  |
| Others | 1 (0.42%) | 53 (0.19%) |  |
| Anesthesia |  |  | 0.93 |
| Local | 29 (12.2%) | 3500 (12.3%) |  |
| Sedations | 0 | 51 (0.18%) |  |
| General |  |  |  |
| TIVA | 79 (33.3%) | 9302 (32.7%) |  |
| Inhalation | 129 (54.4%) | 15620 (54.9%) |  |
| Induction agent* |  |  |  |
| Propofol | 78 (60.5%) | 14940 (95.7%) | <0.001 |
| Midazolam | 19 (14.7%) | 1389 (8.9%) | 0.021 |
| Thiopental | 38 (29.5%) | 103 (0.66%) | <0.001 |
| Ketamine | 14 (10.9%) | 861 (5.5%) | 0.008 |
| Others | 2 (1.6%) | 137 (0.88%) | 0.42 |

ASA-PS, American Society of Anesthesiologists physical status; TIVA, total intravenous anesthesia.

The “n” in this table was calculated on the basis of the number of anesthesia records.

*For those with volatile anesthetics for maintenance. Some patients used multiple induction agents.
